# The Enigma of Low-Density Granulocytes in Humans: Complexities in the Characterization and Function of LDGs during Disease

**DOI:** 10.3390/pathogens10091091

**Published:** 2021-08-27

**Authors:** Brittany G. Seman, Cory M. Robinson

**Affiliations:** 1 Department of Microbiology, Immunology, and Cell Biology, West Virginia University School of Medicine, Morgantown, WV 26506, USA; brittany.seman@hsc.wvu.edu; 2 Vaccine Development Center, West Virginia University, Morgantown, WV 26506, USA

**Keywords:** low-density granulocyte, neutrophil, myeloid-derived suppressor cell, infection, cancer, autoimmunity

## Abstract

Low-density granulocytes (LDGs) have been characterized as important immune cells during healthy and disease states in humans, including microbial infections, cancer, and autoimmune dysfunction. However, the classification of this cell type is similar to other immune cells (e.g., neutrophils, myeloid-derived suppressor cells) and ambiguous functional standards have rendered LDG identification and isolation daunting. Furthermore, most research involving LDGs has mainly focused on adult cells and subjects, leaving increased uncertainty surrounding younger populations, especially in vulnerable neonatal groups where LDG numbers are elevated. This review aims to bring together the current research in the field of LDG biology in the context of immunity to disease, with a focus on infection. In addition, we propose to highlight the gaps in the field that, if filled, could improve upon isolation techniques and functional characterizations for LDGs separate from neutrophils and myeloid-derived suppressor cells (MDSCs). This will not only enhance understanding of LDGs during disease processes and how they differ from other cell types but will also aid in the interpretation of comparative studies and results with the potential to inform development of novel therapeutics to improve disease states in patients.

## 1. Introduction

Low-density granulocytes (LDGs) are characterized as important immune cells during healthy and disease states in humans, with elevated numbers occurring in numerous microbial infections, cancers, and autoimmune disorders [1,2,3,4]. Many studies working with LDGs within these disease states classify LDGs as granulocytic myeloid-derived suppressor cells (gMDSCs) and low-density neutrophils (LDNs), with similar cell surface marker expression and functional characterizations utilized for each cell type. Thus, the classification of LDGs through surface marker expression and functional mechanisms remains challenging, especially when distinguishing from neutrophils. Further, MDSCs in recent years have been functionally characterized by their ability to suppress T cell proliferation. However, a set threshold of suppression has not been established in the field, leaving numerous studies with inconsistent suppression levels, which are at times even undetected [5,6,7]. We and others have adopted the strategy of considering cells in the low-density fraction that fail to robustly suppress T cell proliferation as LDGs or LDNs, but this practice is not universally accepted. For the purpose of this review, we describe the cells as the authors of the published work did, even if there is reason to question that classification.

In addition to the classification and functional challenges, LDG research has mainly focused on adult cells and subjects. This has left increased uncertainty in fields surrounding younger populations, especially in vulnerable neonatal groups where LDG/gMDSC numbers are elevated even without disease [8]. Overall, the goal of this review is to bring together the current research in the field of LDG biology, with a main focus on LDGs during infection, alongside comparisons in both cancer and autoimmune disease. We highlight discrepancies in the field that, if improved upon, could enhance isolation and functional characterizations for LDGs independent of neutrophils and myeloid-derived suppressor cells (MDSCs). These improvements will clarify the importance of each cell type during disease states, aid in the comparison of published studies, and in the end could lead to improved therapeutic development and human health.

## 2. Body

### 2.1. Classification Issues

The term “low-density granulocyte” is used to describe a heterogeneous population of granulocytic myeloid cells isolated from the peripheral blood mononuclear layer (PBMC) after density gradient centrifugation. Ficoll^®^ Paque, Lymphoprep^TM^, Histopaque^®^, Percoll^®^ Plus and other media made of polymers suspended in aqueous solutions at varying densities (usually starting at 1.077 g/mL) are largely used to separate LDGs from normal- or high-density neutrophils in whole blood or buffy coats [9,10]. When density gradient media are layered appropriately under or above diluted whole blood/buffy coats and centrifuged without a brake for acceleration or deceleration, a gradient will form. In the simplest example, the gradient will yield heavier granulocytes and erythrocytes in a pellet at the bottom of the tube, a density media layer, and a top layer that includes mononuclear cells and other low-density cell types [11]. Once the mononuclear cells are separated, LDGs/low-density neutrophils (LDNs)/granulocytic MDSCs (gMDSCs) can be further isolated using cell surface marker expression via cell sorting or magnetic bead isolation.

In our most recent publication, we describe our neonatal LDGs (from cord blood mononuclear cells, or CBMCs) as CD66^hi^, CD33^+^, CD14^lo^, and HLA-DR^−^, a phenotype established in a 2013 report on neonatal gMDSCs by Rieber et al. [3,7]. Other studies have used similar cell surface markers to characterize LDGs, gMDSCs, and LDNs (Table 1). He et al. [8] described gMDSCs from both adult and neonatal patients with CD11b^+^, CD14^−^, and CD15^+^ markers. However, in adults infected with *Mycobacterium tuberculosis* (Mtb), LDNs were characterized as CD33^+^, CD66^+^, CD11b^+^, and HLA-DR^+^ [2]. The expression of HLA-DR in this study in comparison to other studies which cite a lack of HLA-DR expression on LDGs highlights one inconsistency in surface marker expression as a means of LDG classification, although the level of expression was unimpressively different from the isotype control. Another well-known surface marker for LDGs is CD33, which is positively expressed in most studies on gMDSCs, LDGs, and LDNs (Table 1). However, in some studies of cancer, autoimmunity, and healthy donors, CD33 expression alters between moderate and low expression [12,13,14]. The discrepancies noted with HLA-DR and CD33, and other cell surface marker discrepancies, lead us to hypothesize that LDG surface marker expression (and potentially immune function, reported later in this review) could be age, microbe, and pathologically context-dependent. In addition, the elapsed time between blood collection from patients and isolation of cells, potentially reducing or eliminating disease-associated factors or signals, could also alter the expression of some cell surface markers. For instance, CD66 is found abundantly on both normal-density neutrophils (NDNs) and LDGs/LDNs/gMDSCs [15,16] (Table 1). However, there is some evidence that CD66 expression is increased further after NDNs have undergone degranulation, effectively altering their buoyancy status to a lower density [17,18,19,20]. Degranulation can occur excessively in many inflammatory disease states, including autoimmune disorders and sepsis [21]. Overall, the discrepancies in expression described here for surface markers serve as indicators that we should reconsider how we classify and identify LDGs. For instance, should both HLA-DR and CD33 be considered markers for use in determining LDGs from other cell types, or is there a potential threshold of each marker (e.g., medium to high expression of CD33, or low to negative expression of HLA-DR) that should be considered when naming LDGs?

Several surface markers are implemented in the global phenotyping of all three granulocytic cell types: positive expression of CD11b, CD15, and/or CD66 (Table 1). With similar expression levels across LDGs, LDNs, and gMDSCs, as well as NDNs, a question arises: if these cells are different from one another, how do we separate them beyond surface marker expression? Some studies in lupus, cancer, and other diseases have suggested the additional marker of LOX-1 to further clarify LDGs/gMDSCs apart from NDNs, though this has not become a regular component of the classification, and perhaps more importantly, it is unclear if it can distinguish LDGs/LDNs from gMDSCs in the same mononuclear fraction [6,22]. Could and should LOX-1 become a novel marker to further clarify LDGs? A separate study involving lupus LDGs described subsets of LDGs based on CD10 surface expression: CD10^−^ LDGs were classified as immature LDGs that were unable to synthesize inflammatory damage on endothelial tissues and did not phagocytose bacteria or release significant levels of MPO compared to CD10^+^ LDGs, which had enhanced chemotactic and type I IFN-stimulated gene expression with lower levels of cell cycle-related genes [23]. Could CD10 be another marker that could be used to differentiate LDGs from gMDSCs? These questions must be addressed to further prevent confusing interpretations and inappropriate comparisons of published findings in the current literature.

### 2.2. Functional Issues

For many years, gMDSCs have been characterized by their ability to suppress T and natural killer (NK) cell function through their release of arginase-1 (ARG-1), inducible nitric oxide synthase (iNOS), tumor growth factor beta (TGFβ), and other effector molecules [29,56,57,58,59]. Suppression of T cell proliferation has indeed become a functional hallmark for MDSC assignment, but there are lapses in demonstration of this characteristic that are found throughout the literature. In addition, recent work in our lab has shown that LDGs inhibit innate immunity, specifically some aspects of monocyte killing of bacteria during co-culture [3]. This finding is consistent with the release of extracellular DNA from LDGs, and the inclusion of DNAse during co-culture increases bacterial viability [3]. Cell-free conditioned media from LDG cultures further impaired monocyte phagocytosis of bacteria. This work suggests that release of DNA could be considered an effector mechanism for immune suppression [3]. Dietz et al. also demonstrated suppression of innate immunity with cells they describe as gMDSCs [35]. In their study, gMDSCs impaired the ability of monocytes to stimulate T cell proliferation, as well as inhibiting the ability of monocytes to phagocytose bacteria. This group demonstrated induction of inhibitory molecules by gMDSCs that decreased MHC class II, CD11b, and CD18 markers on monocytes [35]. Collectively, these studies highlight immunosuppressive activity associated with gMDSCs and LDGs.

While immune suppressive activity is widely associated with gMDSCs and LDGs/LDNs, numerous studies have recently appeared that detect varying degrees of immune suppression on T cells specifically, including no suppressive activity at all (either unexamined or undetected), that signals a need for guidance on the interpretation of immunosuppression as a functional characteristic of MDSCs versus LDGs. In a study involving LDNs from patients suffering from chronic graft-versus-host disease, Matthews et al. discovered that this cell type enhanced T cell proliferation [5]. LDGs from patients with SLE (systemic lupus erythematosus) induced T cells to produce significantly higher levels of interferon gamma (IFN-γ) and tumor necrosis factor alpha (TNF-α), two proinflammatory cytokines, compared to NDNs [6]. In our recent study, we found that neonatal LDGs only moderately suppressed T cell function, at proliferation indices of 0.75 for CD8^+^ cells and 0.62 for CD4^+^ cells [3]. In another study using neonatal gMDSCs, T cell proliferation indices were much lower (e.g., ~0.3 for CD8^+^ cells, ~0.4 for CD4^+^ cells) [7]. In adults with head/neck or urologic cancer, Lang et al. found that proliferation of T cells was significantly reduced in the presence of gMDSCs, though some of the individual data points graphed suggest that there were at least some patients in which T cell proliferation was not affected by gMDSCs [60]. In a recent study on the effect of gMDSCs during SARS-CoV-2 infection, Agrati et al. found that adding gMDSCs at a fivefold ratio to T cells reduced proliferation from 50.6% to 34.5% [61]. It is reasonable to question the biological relevance of this ratio and it does speak to limited suppressive activity on a per cell basis. These data overall suggest that there is no clear interpretation for the amount, or threshold, of suppression in the functional characterization of LDGs/LDNs/gMDSCs. In addition, there seems to be no clear indication of the amount of LDGs/LDNs/gMDSCs that are necessary for T cell suppression (e.g., 1:1 ratio of LDGs:T cells versus 5:1 ratio of gMDSCs:T cells). Thus, we suggest that a functional standard for suppression of T cells be established for classification of granulocytic cells. For instance, is a threshold of 50% suppression of T cell proliferation sufficient for considering cells gMDSCs? Is there a specific threshold of suppression that must occur before an LDG/LDN is considered a gMDSC, and what are the details of the assay to be used? If not T cell suppression, is some other functional characteristic worthy of being used? These functional thresholds should then be applied to LDGs and gMDSCs from different age groups and disease states to further clarify the cell type under investigation.

### 2.3. Disease States and Issues

#### 2.3.1. LDGs during Infection

Recent work has expanded on the increasing importance of LDGs during infection. LDG/gMDSC populations are increased during mild and severe SARS-CoV-2 [61,62,63], bacterial sepsis [64], HIV/AIDS [65], and tuberculosis (Mtb) [66], among other infections. A major objective of these studies is to determine whether the increased abundance of LDGs is consistent with a more severe pathological outcome. Overall, Janols et al. found that gMDSCs were more abundant in patients with both Gram-positive and Gram-negative bacterial sepsis infections [64]. In addition, patients with elevated numbers of gMDSCs early during sepsis were more susceptible to severe infections and had a higher probability of acquiring secondary hospital infections [67]. Patients with HIV infection were also found to have elevated gMDSCs, which correlated with increasing viral load and decreasing numbers of CD4^+^ T cells, suggesting that gMDSCs may suppress T cell proliferation during HIV infection [68]. Indeed, MDSCs from HIV-infected progressors restricted proliferation of CD8^+^ T cells from healthy donors and HIV-controllers in vitro [68]. The cystic fibrosis opportunistic pathogen *P. aeruginosa* not only increases gMDSC abundance during infection, but this gMDSC increase also seems to correlate with overall lower lung function in juvenile patients [31]. In adult patients with Mtb, gMDSCs were present in higher levels in both the lungs and peripheral blood compared to healthy controls, and this expansion correlated with a significant increase in nitric oxide in the blood [66]. Importantly, arginine levels were significantly depleted in Mtb patients, suggesting that gMDSCs, which produce nitric oxide synthase, may be metabolizing arginine to generate nitric oxide for inhibition of T cell proliferation [66]. These studies strongly suggest that increased LDG levels correlate with poor outcomes during infection.

We and others have found that LDGs and gMDSCs can phagocytose bacteria during infection. In our recent publication, we examined the phagocytic function of neonatal LDGs compared to monocytes from the same cord blood mononuclear cell (CBMC) layer, and found that, although less efficiently than monocytes, LDGs are able to phagocytose pathogenic *E. coli*, traffic the bacteria into acidic lysosomes, and eventually kill the bacteria [3]. Lieber et al. also found that neonatal gMDSCs phagocytosed a laboratory strain of *E. coli* and Group B *Streptococcus* at similar rates compared to neonatal NDNs [34]. LDGs are also noted to phagocytose the malaria-causing parasite *P. vivax* similarly to NDNs in afflicted adult patients [43]. In a recent report, adult LDNs were shown to phagocytose opsonized latex beads at a significantly higher rate than NDNs [49]. We also demonstrated similar findings in that the frequency of neonatal LDGs capable of phagocytosing large numbers of pathogenic *E. coli* O1:K1:H7 is greater than neonatal NDNs [3]. However, in another study focusing on the effects of LDNs in Mtb patients, La Manna et al. found that these cells did not phagocytose Mtb compared to NDNs [2]. The disparities in internalization may suggest that the nature of the particle, bacterium, and/or the population from which the cell was derived (neonate versus adult) contributes to the level of activity. Our LDGs also produce extracellular DNA traps (ETs) in the presence of *E. coli* during infection, which is in contrast to the work by La Manna et al. in the context of Mtb infection, where no ETs were produced by LDNs [2]. However, Blanco-Camarillo et al. reported significant release of DNA following LDN stimulation with phorbol myristate acetate (PMA), a mitogen used often in neutrophil activation [49,69]. Overall, these results suggest that there is potentially an age- and microbe-dependent influence on the phagocytic and extracellular DNA trap functionality of LDGs.

Contrary to the hypothesis that increased LDGs equate to worse infectious outcomes in the host, some studies suggest that the presence of some proportion of LDGs may be beneficial. Stoll et al. describe the inhibition of MDSC expansion in the presence of increasing *S. aureus* enterotoxin production [70]. This could promote T and NK cell activity and a stronger inflammatory response to *S. aureus*. However, a study in mice suggests that too many CD4^+^ T cells can actually result in exacerbated infection and damage to tissues during *S. aureus* pneumonia [71], though this has not been shown directly in humans yet. NK cells can also contribute to excessive inflammatory cytokine production during malaria infection that results in increased systemic symptoms, such as fever in humans, and cerebral damage in mice [72,73,74]. Overall, these studies suggest that in some infectious scenarios, LDGs and MDSCs can regulate pathological inflammation and promote a more balanced immune response. Indeed, this balance has been proposed in the fetal-maternal interface (where gMDSCs are elevated compared to non-pregnant women), in which elevated levels of gMDSCs (and monocytic MDSCs) may prevent maternal immune response to paternal antigens and promote successful pregnancy [75]. Similarly, an increased abundance of MDSCs alleviated pathology in a newborn mouse model of necrotizing enterocolitis [76]. This suggests that MDSCs may protect the host from pathological inflammatory responses during the first days of commensal colonization following birth. However, MDSC or LDG activity may counteract protective host responses to pathogens and promote susceptibility to infection by the newborn, a time period in which LDGs are significantly increased compared to older infants, juveniles, and adults [7,8,33,77,78]. Thus, the balance of MDSCs, LDGs, or LDNs may be beneficial or deleterious depending on the circumstances. It is important to note that the increase in LDGs is not the only dysfunction that occurs in the immune systems of newborns; other cells, such as neonatal neutrophils that have impaired migration functions, or macrophages that have impaired cytokine expression, can also affect the immune response of the neonate to infection [79,80]. However, elevated LDG levels in the neonate have been shown in several studies to contribute to impaired phagocytosis of bacteria, inhibition of monocytes to phagocytose bacteria, and an induction in Th2 and T regulatory responses that can promote susceptibility to infection [3,32,35]. Therapeutic manipulation of MDSCs, LDGs, or LDNs during infection or other clinical conditions, especially in neonates, therefore may promote an improved immunological balance.

#### 2.3.2. LDGs during Cancer

LDGs and gMDSCs are well-known cell types found in high abundance during cancer, usually within tumors, though some sources describe monocytic MDSCs as more common within tumors [81]. Elevated levels of MDSCs in association with tumors have been correlated with higher rates of mortality in patients [82]. In patients with non-small cell lung cancer, gMDSCs were elevated in lung tumor samples, and these levels were elevated further as severity of disease progressed from stage I to stage III [27]. Patients with squamous cell carcinoma of the larynx/oropharynx (head and neck cancer) had significantly elevated levels of gMDSCs in tumors compared to healthy donors, and this elevated state correlated with worsened outcome [26]. High levels of gMDSCs (and monocytic MDSCs) also correlated with higher mortality in patients with metastatic colorectal cancer [83]. Thus, the general consensus currently theorized in the cancer field is that the increased presence of LDGs and gMDSCs leads to a poor outcome in patient survival or remission.

LDGs are generally characterized by their ability to suppress T cell function in cancer. However, some studies identify cancer patients with gMDSCs that fail to restrict T cell proliferation. Alfaro et al. has demonstrated that gMDSCs from patients with advanced lung and prostate cancer do not reduce T cell proliferation [25]. Interestingly, the monocytic MDSCs in this study were found to suppress T cell proliferation. In addition, gMDSCs from the cancerous patients produce extracellular traps when exposed to lipopolysaccharide or IL-8, similarly to NDNs from healthy patients [25]. Are these data an indication that these cells are perhaps not true MDSCs? More rigorous classification standards could help clarify these identity discrepancies as they appear, especially when the cells are available in the blood (versus tissue) for additional study.

One major element abundantly explored in cancer patients are therapeutics that target LDG populations. Pharmaceuticals, including maraviroc, gemcitabine, cisplatin, tadalafil, entinostat, and docetaxel, among others, have been studied extensively in both mice and in human clinical trials, with some success [84]. Tasquinimod, an anti-angiogenic chemotherapeutic, has been shown to decrease human prostate cancer tumor growth by 50% compared to controls in a xenograft mouse model [85]. This chemotherapeutic has been shown to directly target MDSC populations in murine prostate cancer models, though clinical trials are still in early phases for determining whether tasquinimod can work as a suppressor of MDSCs in cancer patients [86,87]. Sunitinib, a receptor tyrosine kinase inhibitor that blocks platelet-derived growth factor receptor (PDGF-R), colony-stimulating factor-1 receptor (CSF-1), and vascular endothelial growth factor receptor (VEGF-R), has been used in patients experiencing renal cell carcinoma [88]. Shortly after its introduction as a cancer therapeutic, studies showed that levels of gMDSCs decreased in patients following 1–2 cycles of treatment [89]. However, in a mouse study on the effects of sunitinib in prostate cancer, Fu et al. surprisingly found that sunitinib treatment actually increased the abundance of gMDSCs in peripheral blood of mice, with little effect on the recruitment of gMDSCs to tumor cells [90]. Although these inconsistencies are surprising, differences in therapeutic outcomes between humans and mice are not unprecedented [91,92,93]; thus, there may be key elements of the murine studies that are not translatable. Interest in programmed cell death protein 1 (PD-1) and its ligand (PD-L1) on the surface of gMDSCs has also grown extensively over the years as therapeutic targets [94]. In one study utilizing gMDSCs from patients with non-small cell lung cancer, Kim et al. found that in patients who did not respond to anti-PD-1-therapy (using nivolumab), a higher number of gMDSC-associated chemokines (including CXCL2, CCL23, and CX3CL1) were increased compared to patients who responded to treatment [95]. Accordingly, patients who responded to nivolumab tended to have lower numbers of gMDSCs; surprisingly, this response to nivolumab also tended to exhibit a higher number of T regulatory (T_reg_) cells in peripheral blood. An inverse correlation between T_reg_ cells and gMDSCs was observed in the treatment responsive cohort [95]. The calculated ratio of T_regs_ to gMDSCs predicted treatment responsiveness when it exceeded a threshold value of 0.39 [95]. Indeed, a better prognosis in non-small cell lung cancer patients utilizing anti-PD-1 therapy (via pembrolizumab or nivolumab) correlated with lower levels of gMDSCs (and monocytic MDSCs and CD39^+^ CD8^+^ T cells) [96]. Although this section gives a brief overview of LDGs in cancer and some therapeutic advances in the field that could potentially be applied to infection studies, there are still many additional studies to be conducted. Most of these studies describe LDGs as gMDSCs, which seems to be a general consensus among the cancer field, save for a few studies using the nomenclature LDN (Table 1). This reasserts the question in the cancer field, are all gMDSCs from cancer patients equivalent to LDGs, or is there sufficient heterogeneity that both populations with differential phenotypic and functional characteristics exist? We suggest a need for single cell sequencing-based studies that are paired with functional assays using low-density cell granulocyte fractions from cancer patients to better discern the differences between gMDSCs and LDGs in this field.

#### 2.3.3. LDGs in Autoimmunity

Low-density granulocytes are almost synonymous with diseases such as lupus (SLE) in the autoimmune field since Hacbarth et al. discovered PBMCs from SLE patients “contaminated with low buoyant density neutrophils” [97]. In this disease, LDGs do not suppress T cell proliferation but rather induce inflammatory cytokines from T cells, including IFN-γ and TNF-α [6], a sharp contrast from other studies in cancer and infection. This inflammatory increase exacerbates SLE and can lead to other inflammatory issues including vascular inflammation and atherosclerosis of the heart [98,99]. LDGs in SLE also produce extracellular traps of DNA that induce damage to endothelial cells, potentially through mitochondrial reactive oxygen species generation [99,100]. Mistry et al. performed an in-depth transcriptomic, chromatin accessibility, and functional analysis of CD66^+^ neutrophil subpopulations. This included two separate LDG populations that were CD10^−^ and CD10^+^, as well as CD10^+^ NDNs from lupus patients [23]. The CD10^−^ LDGs were regarded as more immature, restricted proliferation and IFN-γ production from T cells, and were impaired in their ability to generate NETs, phagocytose bacteria, and release MPO compared to CD10^+^ LDGs [101]. Both subsets were also elevated in lupus patients, but only CD10^+^ LDGs correlated with increased organ damage, including renal failure [23]. Interestingly, CD10^−^ LDGs, CD10^+^ LDGs, and CD10^+^ NDNs could be discriminated based on transcriptional profile, with the CD10^−^ LDG fraction expressing the lowest levels of type I IFN-stimulated genes, as well as a set of other genes associated with the cell cycle, phagocytosis, and chemotaxis [23]. How do we characterize these LDGs across fields where this nomenclature and others are used (gMDSC, LDN)? It is tempting to speculate that CD10^−^ LDGs, CD10^+^ LDGs, and CD10^+^ NDNs may correspond to gMDSCs, LDNs, and NDNs, respectively. Further studies with healthy and other disease states will be required to know whether or not this is the case. This study by Mistry et al. further suggests that within a population of LDGs from lupus patients, the threshold of expression of specific genes defines subpopulations of LDGs that align with functional outcomes during lupus, such as organ damage and inflammation [23]. Could further subcategorization of LDGs from both cancer and infection help us to characterize LDGs, as is seen in Mistry et al., and could a gene expression pattern along with cell surface marker phenotyping and functional activity unlock a universal classification formula?

Alongside their role in inflammation in SLE patients, LDGs may have a role in vasc+ular inflammation in anti-neutrophil cytoplasm autoantibody (ANCA) vasculitis (AAV). LDGs are elevated in AAV patients compared to healthy patients. However, these cells are not responsive to anti-MPO antibodies, and were unable to produce high levels of ROS with antibody compared to NDNs [37], suggesting that these LDGs may be affecting more than just inflammation during this disease. LDG levels are also elevated significantly in patients with moderate-to-severe asthma [102] and may correlate with decreased lung function during disease. In adult and juvenile autoimmune arthritis (adult Still’s disease, or juvenile idiopathic arthritis, JIA), LDGs are elevated in peripheral blood compared to healthy donors [103,104]. Interestingly, Ramanathan et al. found that expression of some neutrophil granule proteins, such as elastase and MPO, was significantly elevated in JIA patients compared to healthy controls, which correlates with the increased extracellular trap production and corresponding toxicity often seen in autoimmune dysfunction patients [103]. Extracellular trap formation and DNA-elastase complexes were also significantly elevated in adult patients compared to healthy controls [104]. Elevated levels of LDGs and subsequent extracellular trap formation are also observed in patients with another form of autoimmune arthritis called pyogenic arthritis, pyoderma gangrenosum and acne (PAPA) syndrome [45]. LDGs have also been found in elevated states in patients with both multiple sclerosis (MS) and neuromyelitis optica spectrum disorder, both of which are neuroautoimmune diseases, compared to healthy controls [105]. In particular, MDSCs as a whole have also been found to be elevated in relapsing-remitting MS patients, though monocytic MDSCs represented the majority of the population elevation [106]. However, it is currently unknown whether these cells were LDGs but perhaps inappropriately named MDSCs during the study, considering that CD3^+^ T cells actually increased in proliferation in cultures with the MDSCs [106]. Thus, elevated LDGs in autoimmune disease can be found systematically (e.g., SLE), or limited to organ-specific populations, such as the neuroautoimmune disorders described above. Overall, the prevalence of LDGs in various forms of autoimmune disease, ranging from lupus to asthma to arthritis, seems to be associated with inflammation and exacerbated disease onset. As stated earlier, this correlation seems to be in stark contrast to the suppressive effects that we normally associate with LDGs during infections and cancer. With this statement, it is important to ask: are these LDGs really LDGs or could they be another associated cell type? If these cells are LDGs, then how do we adjust our thinking on what we call gMDSCs, LDNs, and LDGs during infections and cancer?

## 3. Conclusions

**Figure 1:**
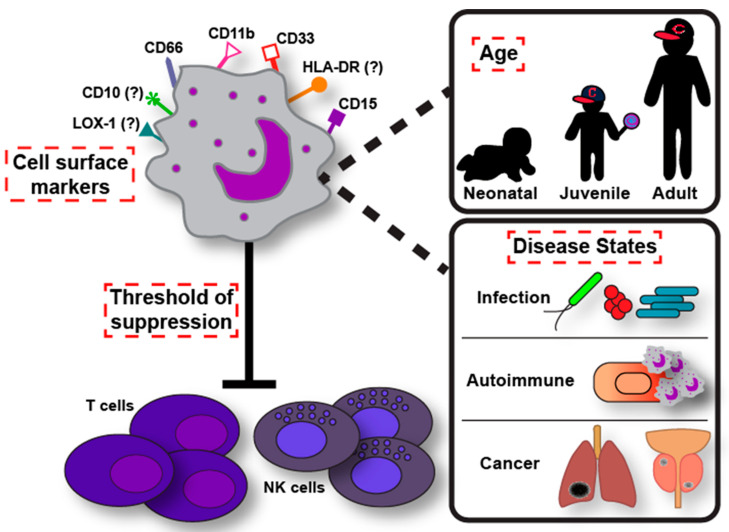
What defines a low-density granulocyte? This schematic depicts an LDG and four of the categories of conflict that we discuss in this article, outlined in red: cell surface marker expression, a threshold of suppression for T cell/NK cell proliferation, the differences in LDG abundance in different age groups, and the functional differences that occur in different disease states, including microbial infections, autoimmunity dysfunction, and cancer. We highlight the need for a better classification of LDGs, distinct from gMDSCs. Cell surface markers, especially CD66, CD11b, and CD15, are common among LDGs, gMDSCs, and LDNs, while some markers, such as HLA-DR, CD33, LOX-1, and CD10 have been suggested as classifying markers, although differences in expression of these markers occur dependent on the study. In the context of T cell and NK cell suppression, we suggest a threshold of suppression (perhaps at least 50% or more) of T cells (and potentially NK cells) with a standard assay to classify cells as gMDSCs apart from LDGs/LDNs. It is important to note that LDG/gMDSC abundances differ depending on the age of the individual (e.g., higher abundance of these cell types in neonates), as well as the disease state the individual is in (e.g., higher abundance of these cell types in tumors, lupus, and microbial infections compared to healthy individuals). Both age and disease state should be taken into consideration when classifying LDGs so that a more clarified determination of therapeutic targeting can occur. Infections associated with LDGs/gMDSCs include Gram-positive bacteria (e.g., *S. aureus*, red), Gram-negative bacteria (e.g., *E. coli*, green), and mycobacteria (e.g., Mtb, blue). Autoimmune disease is depicted as LDGs attacking a host cell and causing inflammation. Cancer tumors are depicted as black spots in example organs, such as the lungs and the prostate.

It is clear from this brief review that there are questions that still must be addressed in regard to the classification of LDGs throughout disciplines. How do we differentiate LDGs from gMDSCs and LDNs? How do we unite as a field to clarify our classification of LDGs in different disease states and within different age ranges? Figure 1 depicts some of the questions that still remain in the field to clarify the meaning of “low-density granulocyte”: what cell surface markers should be used? What threshold of immune cell suppression should be used to justify calling these cells LDGs? Finally, how do we bring together the field of LDG biology in infections, cancer, autoimmune disease, and other dysfunctions of the body? Clarifying the specificities (e.g., regulatory targets, surface marker expression, etc.) of gMDSCs and LDGs/LDNs during disease states will be important in the determination of therapeutic targeting. Not only will immunotherapeutic targeting improve, but the downstream benefits of the health and wellness of patients within certain disease states will improve along with our understanding of comparative studies.

## Figures and Tables

**Table 1:** Classification of LDGs, gMDSCs, and LDNs based on disease state, age, and surface marker expression.

Cell Type	Surface Marker Expression	Disease State	Age	Reference
**gMDSC**	CD11b^+^, CD14^−^, CD15^+^, CD66b^+^	Cancer, Breast	Adult	[24]
**gMDSC**	CD33^+^, CD11b^+^, CD15^+^, HLA-DR^−/lo^	Cancer, Prostate, Head and Neck	Adult	[25]
**gMDSC**	LOX-1^+^, CD66b^+^	Cancer, Head and Neck	Adult	[26]
**gMDSC**	HLA-DR^−/lo^, CD11b^+^, CD14^−^, CD15^+^	Cancer, Lung	Adult	[27]
**gMDSC**	CD11b^+^, CD14^−^, CD15^+^, CD33^+^, HLA-DR^−^	Cancer, Multiple Myeloma	Adult	[28]
**gMDSC**	CD11b^+^, HLA-DR^−^, CD33^mo^, CD66b^+^	Cancer, Pancreatic and Gastric	Adult	[12]
**gMDSC**	CD11b^+^, CD15^+^	Cancer, Prostate, Lung, Head and Neck, Breast, Melanoma	Adult	[29]
**gMDSC**	CD66b^+^, CD33^+^, CD14^−^, HLA-Dr^lo/−^	Healthy	Adult	[30]
**gMDSC**	CD15^+^, CD33^low^, HLA-DR^−^	Healthy	Adult	[13]
**gMDSC**	CD33^hi^, CD66b^hi^, IL-4RA^inter^, HLA-DR^dim^	Infection, *P. aeruginosa*	Pediatric	[31]
**gMDSC**	CD66b^+^	Healthy	Neonatal	[32]
**gMDSC**	CD66b^hi^, CD33^hi^, IL-4RA^inter^, HLA-DR^−^	Healthy	Neonatal	[7]
**gMDSC**	CD66b^+^, CD33^+^, CD14^−^, HLA-Dr^lo/−^	Infection, bacterial sepsis	Neonatal	[33]
**gMDSC**	CD66b^+^	Infection, *E. coli*	Neonatal	[34]
**gMDSC**	CD66b^+^	Infection, *E. coli*	Neonatal	[35]
**gMDSC**	HLA-DR^−^, CD14^−^, CD33^+^, CD11b^+^, CD15^+^	Healthy	Neonatal/Adult	[36]
**gMDSC**	CD11b^+^, CD14^−^, CD15^+^	Healthy	Neonatal/Adult	[8]
**LDG**	LDG-A: CD10^hi^, CD11b^hi^, CD16^hi^, CD33^lo^, CD66b^hi^ LDG-B: CD10^−^, CD11b^lo/int^, CD16^lo^, CD33^int^, CD66b^hi^	Autoimmune, ANCA-AAV	Adult	[14]
**LDG**	CD15^+^, CD14^−^	Autoimmune, AAV	Adult	[37]
**LDG**	CD14^+^, CD15^hi^, CD16^lo^	Autoimmune, RA	Adult	[38]
**LDG**	LIN^−^, HLA-DR^−^, CD11b^+^, CD33^+^, CD15^+^	Autoimmune, SLE	Adult	[6]
**LDG**	CD15^+^, CD14^lo^, CD10^+^	Autoimmune, SLE	Adult	[39]
**LDG**	CD15^+^, Cd14^lo^, CD10^+^	Autoimmune, SLE	Adult	[40]
**LDG**	CD11b^+^, CD62L^lo^	Autoimmune, SLE	Adult	[41]
**LDG**	CD11b^+^, CD15^+^, CD16^+^, CD33^+^, CD66b^+^	Infection, HIV	Adult	[42]
**LDG**	CD14^lo^, CD15^+^	Infection, *Mtb*	Adult	[19]
**LDG**	SSC^hi^, CD66b^+^, CD16^+^, CD14^−^, MCHII^−^, CD15^+^	Infection, *P. vivax*	Adult	[43]
**LDG**	CD62L^lo^, CD66b^+^, CD41a^+^	Infection, SARS-CoV-2	Adult	[44]
**LDG**	CD10^−/+^, CD14^lo^, CD15^+^	Autoimmune, PAPA/SLE	Adult/Pediatric	[45]
**LDG**	CD15^+^	Autoimmune, SLE	Pediatric	[46]
**LDG**	CD66^hi^, CD33^+^, CD14^lo^, HLA-DR^−^	Infection, *E. coli*	Neonatal	[3]
**LDN**	CD15^+^, CD66b^+^	Cancer, Gastric	Adult	[47]
**LDN**	CD15^+^, CD11b^+^, CD66b^+^	Cancer, Lung	Adult	[48]
**LDN**	CD10^+^, CD11b^+^, CD14^lo^, CD15^hi^, CD16b^hi^, CD62L^+^, CD66b^+^, CXCR4^+^	Healthy	Adult	[49]
**LDN**	CD16^+^, CD15^+^, CD33^+^, CD66b^hi^, CD114^+^, CD11b^+/lo^	Infection, bacterial sepsis/*E. coli*	Adult	[50]
**LDN**	CD15^+^, CD33^+^, CD66b^+^, CD62L^lo^, CD80^lo^, CD114^lo^, CXCR4^lo^	Infection, HIV	Adult	[51]
**LDN**	HLA-DR^+^, CD66b^+^	Infection, *Leishmania* spp.	Adult	[52]
**LDN**	CD33^hi^, CD14^−^, CD15^+^	Immunodeficiency, CVID	Adult	[53]
**LDN**	CD33^+^, CD66^+^, CD11b^+^, CD10^+^, CD15^+^, CD13^+^, Cd16^+^, HLA-DR^+^	Infection, *Mtb*	Adult	[2]
**LDN**	CD66b^+^, CD16^+^	Infection, SARS-CoV-2	Adult	[54]
**LDN**	HLA-DR^+^, CD66b^+^	Infection, *Leishmania* spp.	Adult/Pediatric	[55]
